# Production and Partial Characterization of an Alkaline Xylanase from a Novel Fungus* Cladosporium oxysporum*

**DOI:** 10.1155/2016/4575024

**Published:** 2016-04-26

**Authors:** Guo-Qiang Guan, Peng-Xiang Zhao, Jin Zhao, Mei-Juan Wang, Shu-Hao Huo, Feng-Jie Cui, Jian-Xin Jiang

**Affiliations:** ^1^School of Food and Biological Engineering, Jiangsu University, Zhenjiang 212013, China; ^2^Department of Chemical Engineering, Beijing Forestry University, Beijing 100083, China; ^3^Beijing Biomass Energy Technology Center, State Grid Energy Conservation Service Ltd., Beijing 100053, China

## Abstract

A new fungus* Cladosporium oxysporum* GQ-3 producing extracellular xylanase was isolated from decaying agricultural waste and identified based on the morphology and comparison of internal transcribed spacer (ITS) rDNA gene sequence.* C. oxysporum* produced maximum xylanase activity of 55.92 U/mL with wheat bran as a substrate and NH_4_Cl as a nitrogen source. Mg^2+^ improved* C. oxysporum* xylanase production. Partially purified xylanase exhibited maximum activity at 50°C and pH 8.0, respectively, and showed the stable activity after 2-h treatment in pH 7.0–8.5 or below 55°C. Mg^2+^ enhanced the xylanase activity by 2% while Cu^2+^ had the highest inhibition ratio of 57.9%. Furthermore,* C. oxysporum* xylanase was resistant to most of tested neutral and alkaline proteases. Our findings indicated that* Cladosporium oxysporum* GQ-3 was a novel xylanase producer, which could be used in the textile processes or paper/feed industries.

## 1. Introduction

Xylan is wide variety of highly complex polysaccharides mainly composed of xylose units in renewable lignocellulosic biomass resources such as cereal straws, sugarcane bagasse, corn stover, and wood sawdust [1]. Generally, xylan must be firstly hydrolyzed to liberate the fermentable xylose for complete utilization of theses lignocellulosic materials [2]. Microbial or enzymatic hydrolysis of xylan requires the integrative action of multiple enzyme complexes including xylanase (1,4-*β*-D-xylan xylanohydrolase, EC 3.2.1.8), *β*-xylosidase (EC 3.2.1.37), *α*-L-arabinofuranosidases, and *α*-glucosidases [3]. Among them, xylanases have the significant merits of high specificity, mild reaction conditions, and negligible substances loss/side products generation [4, 5] and have been commercially applied in the paper, food additives, feed, and fruit juices industries [6–9].

Xylanases are produced by bacteria [10], fungi [11], actinomycetes [12], and engineered yeasts [13]. Of these, filamentous fungi such as* Aspergillus*,* Penicillium*, and* Trichoderma* have been proved to have a significant capability of secreting a wide range of xylanases. Our previous work also found that the screened fungi* Penicillium* sp. WX-Z1,* Penicillium oxalicum* ZH-19, and* Aspergillus* sp. Zh-26 could produce xylanases with the highest activity of 46.50 U/mL, 80.23 U/mL, and 47.30 U/mL under their optimized fermentation conditions, respectively [14, 15]. For a more efficient degradation of xylan, it is still an ongoing interest to find new xylanases with excellent activity and stability in various reaction conditions. Hence, the present study aimed to isolate the potential xylanase secreting strains, identify the strains using morphology and internal transcribed spacer (ITS) rDNA gene sequence analysis, determine culture conditions for maximum xylanase production, and characterize partially purified xylanase.

## 2. Materials and Methods

### 2.1. Isolation of Xylanase Secreting Microorganisms

For screening xylanase secreting strains, about 1.0 g of decaying wheat straw, collected in the Maoshan Town, Zhenjiang City, Jiangsu Province, was added to the screening media (g/L): KNO_3_ 3.0, K_2_HPO_4_ 0.5, MgSO_4_·7H_2_O 0.5, and KCl 0.5. After being cultured at 30°C for 2-d, the isolate was inoculated on Petri dishes containing (g/L) wheat bran 5.0, KH_2_PO_4_ 0.5, and agar 10.0, at 30°C for 6-d. The developed colonies were seeded to the fermentation media consisting of (g/L) wheat bran 5.0, NH_4_Cl 10.0, KH_2_PO_4_ 1.0, NaCl 1.0, MgSO_4_·7H_2_O 1.0, CaCl_2_·2H_2_O 0.5, and yeast extract 1.0, for determining xylanase production. The chemicals of reagent grade for preparing media or determining xylanase activity were obtained from Sinopharm Chemical Reagent Co., Ltd. (Shanghai, China). One fungal isolate with highest xylanolytic activity was designated as GQ-3 and ultimately chosen for further study.

### 2.2. Phylogenetic Analysis of Fungal Isolate

Genomic DNA of fungal isolate was extracted from fresh cultures and used as template for PCR amplification using a method described previously [16]. The ITS1-5.8S-ITS2 rDNA region of the fungi was amplified by PCR using primer set pITS1 (5′-TCCGTAGGTGAACCTGCCG-3′) and pITS4 (5′-TCCTCCGCTT-ATTGATATGC-3′). PCR amplification conditions were as follows: predenaturing at 95°C for 5 min, 30 cycles of denaturation at 95°C for 1 min; annealing at 55°C for 1 min; and prolonging at 72°C for 1 min, before the extension at 72°C for 10 min and final cooling to 4°C. Electrophoresis was used to examine PCR products in a 0.8% (w/v) agarose gel in 1× TAE buffer (0.4 M Tris, 50 mM NaOAc, 10 mM EDTA, pH 7.8). The amplified DNA fragment was cloned into the pMD18-T vector (Takara, Japan) and its nucleotide sequence was determined using an Applied Biosystems 377B automatic DNA sequencer (Applied Biosystems, Foster, CA, USA). The resulting sequences obtained were queried against the GenBank database using Basic Local Alignment Search Tool (BLAST) program. The neighbor-joining (NJ) method and maximum parsimony (MP) method from the PHYLIP suit program were used for constructing the phylogenetic tree. Bootstrap analysis was used to evaluate the tree topology of the NJ data by performing 1,000 resamplings and marking the branching points. The evolutionary distance matrix was estimated according to Kimura's two-parameter model.

### 2.3. Culture Conditions

The culture seed was prepared by growing fungal cells on PDA slopes at 30°C for 6 d and adjusted the cells concentration to 10^6^ spores/mL with sterile distilled water.

The basal medium for one-factor-at-a-time experiments was composed of (g/L) wheat bran 5.0, KH_2_PO_4_ 1.0, MgSO_4_·7H_2_O 1.0, CaCl_2_·2H_2_O 0.3, and yeast extract 1.0. Effect of carbon sources and nitrogen sources on xylanase production was evaluated by substituting glucose or yeast extract in the basal medium, respectively. Four fermentation temperatures from 25°C to 42°C, and 7 initial pHs from 3.0 to 9.0 were used for selecting the optimal temperature and initial pH with the media containing wheat bran (1.0%, w/v). Fermentation was conducted at 30°C on a rotary shaker (150 rpm) for 5 d if not further specified. The sample was withdrawn at regular intervals and centrifuged at 14,000 rpm (4°C) for 10 min to collect supernatant as the crude enzyme for determining xylanase activity.

### 2.4. Xylanase Activity Assays

The xylanase activity was expressed by release of reducing sugars from oat spelt xylan (1%, w/v) using the dinitrosalicylic acid method [17]. Reaction mixture contained 2 mL of a solution of 1% oat spelt xylan in citrate buffer 50 mM, pH 5.0, and 1 mL of the diluted enzyme. The mixture reacted at 50°C for 30 min. One unit of xylanase was defined as the amount of enzyme required to released 1 *μ*mol of xylose from xylan in 1 min.

Protein concentration was determined using a Bradford Protein Assay Kit (Sangon Biotech, Shanghai, China) with bovine serum albumin as a standard protein. Specific activity was expressed as the number of xylanase units per milligram of enzyme protein.

### 2.5. Preparation of Partial Purified Xylanases

The culture supernatant was concentrated 10-fold by ultrafiltration membrane with molecular weight cutoff (MWCO) of 10 kDa (Millipore, MA, USA) and precipitated with 40% saturation ammonium sulfate. The protein precipitate was dissolved in potassium phosphate buffer solution (PBS), loaded onto a DEAE-Sepharose Fast Flow column (1.2 cm × 60 cm) (GE Healthcare, MA, USA), and step-wisely washed with a discontinuous gradient of PBS, PBS containing 0.3 mol/L NaCl, and PBS containing 0.5 mol/L NaCl. The elution fractions having xylanolytic activity were collected and lyophilized for biochemical characterization.

### 2.6. Effect of pH and Temperature on Partially Purified Xylanase Activity and Stability

The enzymes were mixed with buffers with varying pH at 1 : 10 (v/v) ratio and incubated for 60 min to identify the optimal pH and pH stability. The buffers used were as follows: 0.2 M acetate buffer (pH 3.0–5.0), 0.2 M phosphate buffer (pH 6.0–7.5), and 0.2 M Tris–HCl buffer (pH 7.5–9.0).

The optimum catalytic temperature of partially purified xylanase was selected by determining the enzyme activity in the range of 30–70°C. The temperature stability was determined by measuring the residual xylanolytic activity after being treated with temperatures from 40 to 70°C for 0.0, 0.5, 1.0, 2.0, and 3.0 h.

### 2.7. Effect of Metal Ions on Partially Purified Xylanase Activity

Effect of metal ions on xylanase activity was evaluated by incubating reaction mixtures containing 1.0% oat spelt xylan with K^+^, Mn^2+^, Na^+^, Mg^2+^, Fe^2+^, Cu^2+^, Li^+^, Co^2+^, Hg^2+^, and Zn^2+^ at the concentrations of 1.0 mM. Inhibition/activation degree of xylanase activity was expressed as percentage of a control sample incubated in absence of any additive.

### 2.8. Effect of Proteases on Xylanase Activity

Resistance of partially purified xylanase to proteases was tested by adding neutral and alkaline proteases including proteinase K (from* Tritirachium album*), pepsin, trypsin, and collagenase (from* Clostridium histolyticum*) with unit of 10.0 *μ*g/mL. The residual xylanolytic activity was measured after 3-h preincubation at 37°C. The reaction mixture without proteases was used as control.

### 2.9. Statistical Analysis

Each experiment was repeated three times using duplicate samples. The results were expressed as means ± standard deviations. Statistical comparisons were made by one-way analysis of variance (ANOVA), followed by Duncan's multiple-comparison test. Differences were considered significant when the *P* values were <0.05.

## 3. Results and Discussion

### 3.1. Identification of Strain* Cladosporium oxysporum* GQ-3

**Figure 1:**
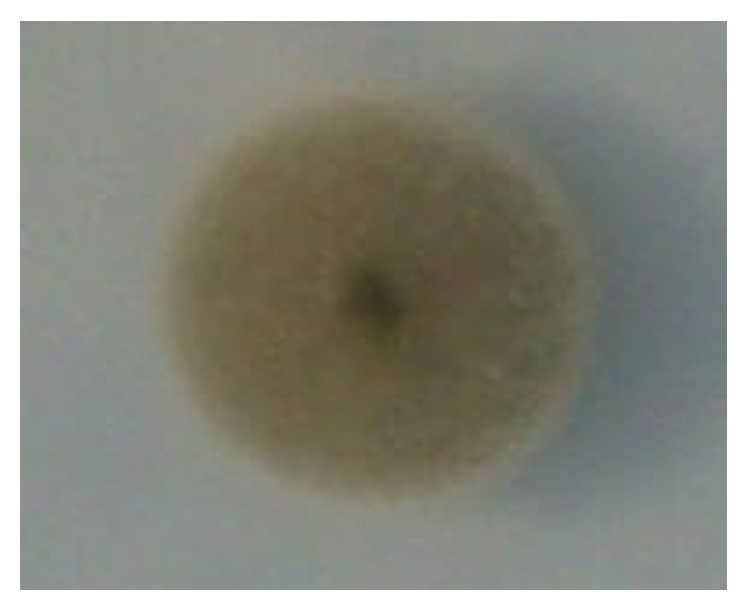
Colony morphology of* Cladosporium oxysporum* after growth at 30°C for 7 days on potato dextrose agar (PDA).

**Figure 2:**
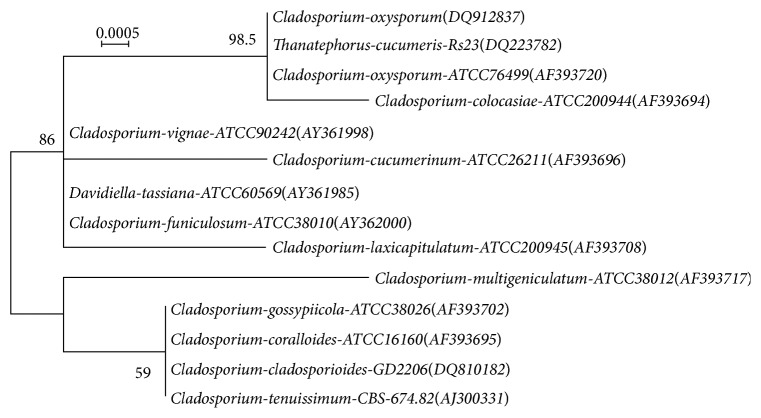
The phylogenetic dendrogram for* Cladosporium oxysporum* and related strains based on the ITS rDNA sequence. Numbers following the names of the strains are accessing numbers of published sequences.

The colony morphology of fungal isolate GQ-3 after 7-d cultivation on potato dextrose agar (PDA) at 30°C was shown in Figure 1. Phylogenetic analysis also confirmed the maximum closeness of the fungal isolate with* Cladosporium oxysporum* (Figure 2). The nucleotide sequence in internal transcribing spacer (ITS) region was also compared with blast (NCBI) and showed 98.5% similarity with that of* Cladosporium oxysporum* ATCC 76499. Hence, the fungal isolate was identified as a strain of* C. oxysporum* and named* C. oxysporum* GQ-3 based on the morphology and comparison of ITS rDNA gene sequence. The sequence has been deposited in NCBI GenBank database with accession number: DQ912837. Previous studies revealed that* C. oxysporum* had the pathogenicity causing death and hyphal growth of* P. citri, Pseudococcus longispinus* (T.T.),* Coccus aethiopicus* De Lotto, and* Trioza erytreae* (del Guercio) [18] and produced the antiproliferative taxol against human pathogenic bacteria and human colon cancer cell line HCT 15 [19]. To the best of our knowledge, the screened* C. oxysporum* GQ-3 was the new record to produce xylanase.

### 3.2. Effect of Carbon Source on Xylanase Production

From Table 1, starch at the concentration of 1.0% (w/v) yielded the low xylanase activity of 1.95 ± 0.46 U/mL while wheat bran led to the maximum xylanase activity of 52.94 ± 3.17 U/mL, followed by oat xylan and corn cob, which indicated that the substrates containing xylan could induce xylanase production by* Cladosporium oxysporum*. Generally, the lignocellulosic material, such as barley husk, corn cobs, hay, wheat bran, or straw, had the advantage of pure xylan or xylose due to the low cost to produce xylanase in a larger scale. Wheat bran is rich in carbohydrates (including glucan 10.5%, xylan 18.3%, and arabinan 10.1%), proteins, fats, and vitamins and has been proved as an effective xylanase inducer. For example, comparing with corncob, cassava bran, corn straw, or sugar cane bagasse,* Neosartorya spinosa* strain P2D19 produced highest xylanase activity (15.1 U/mL) from wheat bran [20]. Xu et al. also reported that the highest xylanase activity achieved 1245 U/mL at a wheat bran concentration of 70.0 g L^−1^ by* Pseudomonas* sp. WLUN024 [21].

Xylose has also been proved as a xylanase inducer in some organisms such as* Aureobasidium pullulans* and* Trichosporon cutaneum*. In the present study,* C. oxysporum* GQ-3 fermented xylose (1.0%, w/v) effectively to produce xylanase of 20.32 U/mL. Other pure sugars such as glucose, fructose, and sobiol were not preferable for* C. oxysporum* with xylanase production of below 3.00 U/mL, which might be due to their prevention of enzyme synthesis [22].

### 3.3. Effect of Nitrogen Source on Xylanase Production

**Figure 3:**
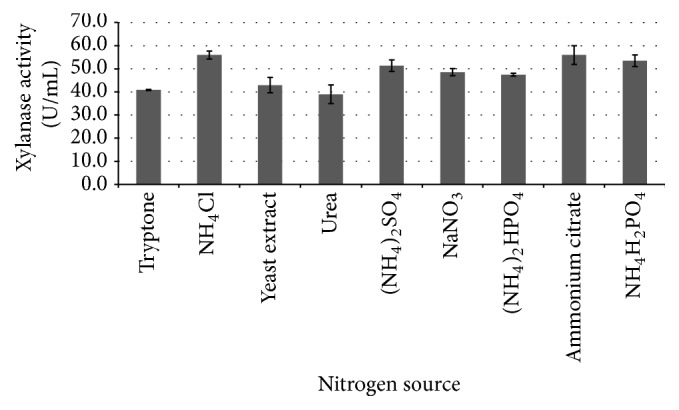
Effect of nitrogen source on xylanase production by* C. oxysporum* GQ-3 at 30°C under submerged fermentation for 5 d (other media compositions/g/L: wheat bran 10.0, KH_2_PO_4_ 1.0, MgSO_4_·7H_2_O 1.0, and CaCl_2_·2H_2_O 0.3).

As shown in Figure 3, organic nitrogen sources at a concentration of 1.0 g/L benefited the xylanase production by* C. oxysporum* GQ3. Tryptone and yeast extract gave the high xylanase activities of 40.82 U/mL and 42.93 U/mL, respectively. Similarly,* Trichoderma harzianum* PPDDN10 NFCCI-2925 produced maximum xylanase activity of 2137.75 IU/gds with mycological peptone (MYP) [23]. In some cases, the inorganic nitrogen sources also benefited the xylanase production by fungi such as* T. harzianum, S. commune, and T. lanuginosus* [23]. In the present study, substitution of yeast extract in the basal media with inorganic nitrogen including NH_4_Cl, (NH_4_)_2_SO_4_, ammonium citrate, and NH_4_H_2_PO_4_ improved xylanase production. The xylanase activity reached the highest level of 55.92 U/mL with NH_4_Cl as a nitrogen source (*P* < 0.05).

### 3.4. Effect of Initial Medium pH and Temperature on Xylanase Production

**Figure 4:**
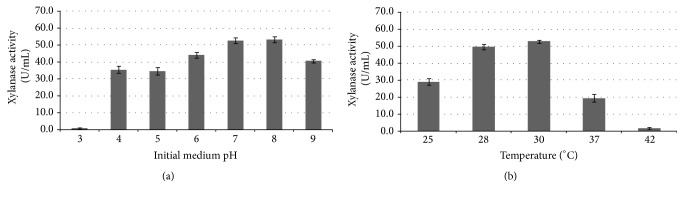
Effect of initial medium pH and temperature on xylanase production by* Cladosporium oxysporum* under submerged fermentation at 30°C (media compositions/g/L: wheat bran 10.0, KH_2_PO_4_ 1.0, MgSO_4_·7H_2_O 1.0, CaCl_2_·2H_2_O 0.3, and NH_4_Cl 1.0).

Generally, initial pH and fermentation temperature were regarded as the significant factors affecting cell growth and xylanase production by influencing nutrients transport and corresponding enzymatic systems. Herein,* C. oxysporum* GQ-3 was cultivated in basal medium at pH values ranging from 3.0 to 9.0 to find the optimal initial pH for xylanase production. As shown in Figure 4(a), lower pH conditions (below pH 6.0) had the negative effect for* C. oxysporum* xylanase production.* C. oxysporum* had a higher xylanase production with the activity of over 44.2 U/mL at slightly acidic pH (pH 6.0), neutral pH (pH 7.0), and slight alkaline pH (pH 7.0–8.0). Our previous study also found that* Penicillium oxalicum* ZH-30 produced the highest xylanase activity of 14.91 U/mL at pH 7.72 [16].

Figure 4(b) presented the influence of temperature ranging from 25°C to 42°C on the xylanase production of* C. oxysporum* with wheat bran concentration of 1.0% (w/v). Temperature from 28°C to 30°C benefited* C. oxysporum* xylanase production with the highest activity of 52.84 U/mL at 30°C. With temperature increase to 42°C, xylanase production decreased significantly to 1.74 U/mL (*P* < 0.05). Nawel et al. also found that temperatures from 30°C to 37°C were suitable for cell growth and xylanase production of* J. denitrificans* BN-13 L [24]. Therefore, 30°C appeared to be the optimal temperature for xylanase production by* C. oxysporum*.

### 3.5. Effect of Metal Ions on Xylanase Production

Fungi require a unique combination of several unusual nutrient conditions, that is, hydrogen ions, dissolved oxygen certain trace metals, or phosphate for sufficient growth or xylanase production.  Table 2 showed effect of 9 metal ions with the concentrations of 1.0 mM or 5.0 mM on* C. oxysporum* xylanase production. Compared with control result, supplementation of Zn^2+^, Fe^2+^, Mg^2+^, Mn^2+^, or Ca^2+^ was positive for xylanase production with activity of over 36.00 U/mL. Mg^2+^ at the concentration of 5.0 mM gave the maximum xylanase activity of 53.04 U/mL. However, Cu^2+^ completely inhibited xylanase production with the activity of 0.24 U/mL (*P* < 0.05). A similar result also was found by Reis et al. with 17% of xylanase activity improvement (30.50 U/mL) after addition of MgSO_4_ and CaCl_2_ [25].

### 3.6. Properties of the Partially Purified Xylanase

#### 3.6.1. Purification of Xylanase from the Fermentation Broth of* C. oxysporum*

Table 3 summarized the* C. oxysporum* xylanase yield and activity during 2-step purification processes. Ammonium sulfate precipitation led to xylanase specific activity of 10.12 U/mg protein and increased purification fold to 3.61. During the DEAE-Sepharose Fast column chromatography, fraction eluted with PBS containing 0.3 mol/L NaCl had the highest xylanase activity of 48.56 U/mL (elution profile not shown). The partially purified xylanase had the increased specific activity of 55.29 U/mg protein and purification fold of 20.98.

### 3.7. Effect of pH on Partially Purified Xylanase Activity

**Figure 5:**
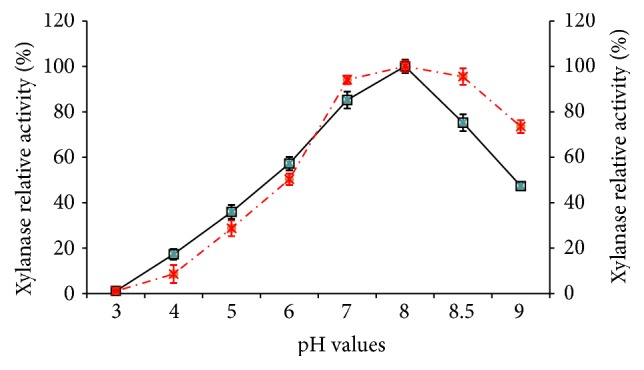
Influence of pH on the activity (-□-) and stability (-**●**-) of xylanase from* C. oxysporum* GQ3 (Media compositions/g/L: wheat bran 10.0, KH_2_PO_4_ 1.0, MgSO_4_·7H_2_O 1.0, CaCl_2_·2H_2_O 0.3, and NH_4_Cl 1.0).

Catalytic pH markedly affects enzymes activity by dissociating bind of substrate and catalysis and even destroying their molecular structures.  Figure 5 presented effect of pH on the xylanase activity and stability. As shown in Figure 5, alkaline condition (7.0–9.0) favored xylanase activity of* C. oxysporum* GQ3. Too high (over 9.0) or low (below 6.0) pH conditions significantly inhibited xylanase activity (*P* < 0.05). Hence, it could be concluded that* C. oxysporum* xylanase could tolerate alkaline conditions and is possibly classified as an alkaline xylanase.  Figure 5 also demonstrated that partially purified* C. oxysporum* xylanase had the optimal activity with the relative value of 100% at pH 8.0. Recently, alkali xylanases attract increasing interest for their applications in paper and feed industries, textile processes, enzymatic saccharification and waste treatments. For example, alkaline xylanase could directly treat the pulp with high pH and avoid the cost/time consuming steps of pH readjustments. Similarly,* Geobacillus thermodenitrificans* also produced an alkali xylanase with the maximum activity at pH 7.5 and 70°C [26].

### 3.8. Effect of Temperature on Partially Purified Xylanase Activity and Thermostability

**Figure 6:**
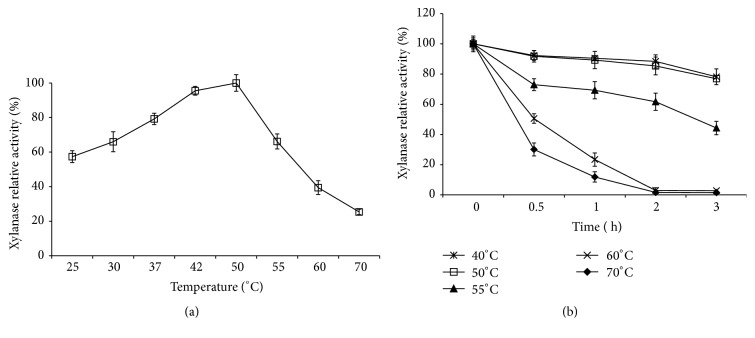
Influence of temperature on the activity of xylanase and its thermostability from* Cladosporium oxysporum* GQ3.

From Figure 6(a),* C. oxysporum* xylanase activity increased from 32.64 U/mL to 56.90 U/mL with increase of temperature from 20°C to 50°C and then decreased to 37.64 U/mL at 50°C and even 14.44 U/mL at 70°C. Hence, the optimum catalytic temperature of* C. oxysporum* xylanase was regarded as 50°C.

*C. oxysporum* xylanase had the stable activity at below 50°C (Figure 6(b)) and approximately 44.30% of its activity remained at 55°C for 3 h. Over 95% of activity was lost after 2-h treatment at over 60°C. These results indicated that 40–50°C was the suitable temperature for future application of* C. oxysporum* xylanase.

### 3.9. Effect of Metal Ions on Partially Purified Xylanase Activity

**Figure 7:**
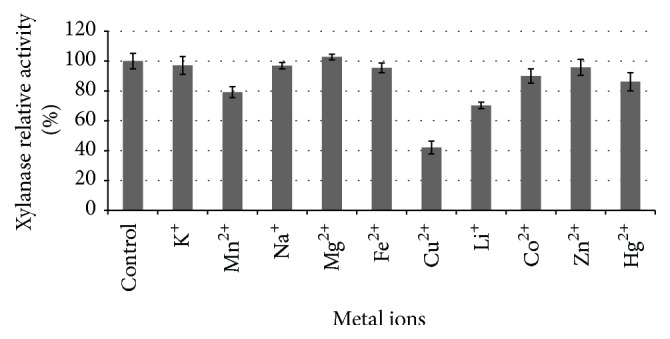
Influence of metal ions on the activity of xylanase from* Cladosporium oxysporum* GQ3.

As shown in Figure 7, most of the metal ions (1.0 mM) had a slight inhibition of about 5.0% on the partially purified xylanase activity while Cu^2+^ had the highest inhibition ratio of 57.9%. Mg^2+^ slightly enhanced partially purified xylanase activity by 2.8%, indicating its possible role as a metal ion cofactor. Previously references have revealed that xylanases activity depended on their producers and metal ion types. For example, Na^+^ and Mn^2+^ strongly inhibited 33.2% and 80.5% of* Alternaria mali* xylanase activity while K^+^, Li^+^, Fe^2+^, Cu^2+^, and Zn^2+^ showed no significant effect [16]. Mn^2+^ significantly inhibited* Streptomyces* sp. xylanase [27], while Hg^2+^ strongly inhibited 66.0% and Ca^2+^ stimulated 51.3% of* C. algeriensis* xylanase [28].

### 3.10. Protease Resistance of Partially Purified Xylanase Activity

The partially purified xylanase from* C. oxysporum* GQ3 showed the resistance to neutral and alkaline proteases when incubated with pepsin, trypsin, collagenase, and proteinase K at 37°C for 3 h. The enzyme retained unchanged activity with collagenase treatment while residual activity was 95.8% and 91.3% of the maximum xylanase activity after the incubation with proteinase K and trypsin, respectively. Interestingly, xylanase produced by* Streptomyces* sp. TN119 GH 11 also exhibited significant re sistance to protease digestion and even increased its activity by 5.8%, 9.4%, 21.6%, and 129.0% after treatments of trypsin, collagenase, *α*-chymotrypsin, and proteinase K, respectively [29].

## 4. Conclusions

In conclusion, the isolated fungus* C. oxysporum* GQ-3 was proved as an efficient xylanase producer with the agroindustrial residue wheat bran as substrate. The* C. oxysporum* partially purified xylanase activity has the optimal catalytic pH and temperature of 8.0 and 50°C, respectively. It showed the pH and thermostable activities while influenced by metal ions. Our study will provide a reference for better understanding of xylanase from* C. oxysporum* and further improving the strain for large scale production and industrial application such as textile processes or paper and feed industries.

## Figures and Tables

**Table 1:** Effect of carbon source on xylanase production by *C. oxysporum* GQ-3 at 30°C under submerged fermentation for 5 d.

Carbon source (w/v)	Xylanase activity (U/mL)^*∗*^
0.5% wheat bran	30.77 ± 1.25
1.0% wheat bran	52.94 ± 3.17
0.5% oat xylan	26.43 ± 1.94
1.0% oat xylan	46.38 ± 2.55
0.5% corn cob	12.66 ± 3.29
1.0% corn cob	33.51 ± 1.48
1.0% starch	1.95 ± 0.46
1.0% glucose	2.82 ± 0.98
1.0% fructose	0.83 ± 0.12
1.0% xylose	20.32 ± 2.41
1.0% sobiol	1.35 ± 0.73
1.0% maltose	2.91 ± 0.22

^*∗*^Other media compositions/g/L: KH_2_PO_4_ 1.0, MgSO_4_·7H_2_O 1, CaCl_2_·2H_2_O 0.3, and yeast extract 1.0.

**Table 2:** Effect of metal ions on xylanase production by *C. oxysporum* GQ-3 at 30°C under submerged fermentation for 5 d.

Metal ions (mM)	Xylanase activity (U/mL)^*∗*^
Control	34.74 ± 1.78
K^+^ (1 mM)	36.78 ± 1.62
Ca^2+^ (1 mM)	36.41 ± 2.11
Ca^2+^ (5 mM)	46.75 ± 1.92
Mg^2+^ (1 mM)	38.39 ± 0.53
Mg^2+^ (5 mM)	53.04 ± 1.53
Fe^2+^ (1 mM)	42.75 ± 1.83
Cu^2+^ (1 mM)	0.24 ± 0.15
Co^2+^ (1 mM)	33.81 ± 0.74
Mn^2+^ (1 mM)	36.32 ± 0.92

^*∗*^Media compositions/g/L: wheat bran 5.0 and yeast extract 1.0.

**Table 3:** Summary of xylanase purification from the fermentation broth of *C. oxysporum*.

Fraction	Total protein (mg)	Total activity (units)	Specific activity (U/mg protein)	Yield (%)	Purification fold
Culture filtrate	1039.05	2737.83	2.63	100.00	1.00
(NH_4_)_2_SO_4_ precipitation	97.56	987.28	10.12	36.06	3.61
DEAE-Sepharose Fast Flow	9.83	543.51	55.29	19.85	20.98
